# Barriers and facilitators to mobile health and active surveillance use among older adults with skin disease

**DOI:** 10.1111/hex.13229

**Published:** 2021-06-30

**Authors:** Austin Johnson, Neha Shukla, Meghan Halley, Vanessa Nava, Janya Budaraju, Lucy Zhang, Eleni Linos

**Affiliations:** ^1^ Department of Dermatology School of Medicine Program for Clinical Research and Technology Stanford University Stanford CA USA; ^2^ School of Medicine Center for Biomedical Ethics Stanford University Stanford CA USA

**Keywords:** active surveillance, ageing, dermatology, mobile health, older adults, telemedicine

## Abstract

**Background:**

The COVID‐19 pandemic has accelerated the adoption of telemedicine, including teledermatology. Monitoring skin lesions using teledermatology may become increasingly important for several skin diseases, including low‐risk skin cancers. The purpose of this study was to describe the key factors that could serve as barriers or facilitators to skin disease monitoring using mobile health technology (mHealth) in older adults.

**Methods:**

Older adult dermatology patients 65 years or older and their caregivers who have seen a dermatologist in the last 18 months were interviewed and surveyed between December 2019 and July 2020. The purpose of these interviews was to better understand attitudes, beliefs and behaviours that could serve as barriers and facilitators to the use of mHealth and active surveillance to monitor low‐risk skin cancers.

**Results:**

A total of 33 interviews leading to 6022 unique excerpts yielded 8 factors, or themes, that could serve as barriers, facilitators or both to mHealth and active surveillance. We propose an integrated conceptual framework that highlights the interaction of these themes at both the patient and provider level, including care environment, support systems and personal values.

**Discussion and conclusions:**

These preliminary findings reveal factors influencing patient acceptance of active surveillance in dermatology, such as changes to the patient‐provider interaction and alignment with personal values. These factors were also found to influence adoption of mHealth interventions. Given such overlap, it is essential to address barriers and facilitators from both domains when designing a new dermatology active surveillance approach with novel mHealth technology.

**Patient or public contribution:**

The patients included in this study were participants during the data collection process. Members of the Stanford Healthcare and Denver Tech Dermatology health‐care teams aided in the recruitment phase of the data collection process.

## 1 INTRODUCTION

Skin cancer is the most common cancer, with over 5 million new cases diagnosed in the United States each year. Basal cell carcinoma (BCC) comprises the majority (80%) of these diagnosed skin cancers.^1^ Over 1 million new cases of BCC in the United States are treated each year in patients over 65, with more than 100 000 treated in the last year of life.^2^, ^3^ Most BCCs grow slowly, rarely metastasizing or affecting quality of life. Nonetheless, the majority of BCCs are treated,^4^, ^5^, ^6^ although up to one‐quarter report complications related to their BCC treatment.^2^, ^7^, ^8^

Active surveillance is used to manage other low‐risk or slow‐growing cancers,^9^ including prostate and thyroid cancer, in which the risks of treatments including surgery or radiation therapy outweigh the benefits.^10^, ^11^, ^12^, ^13^ However, active surveillance has yet to be used in the management of low‐risk skin cancer. Finally, the COVID‐19 pandemic and increasing permanence of telehealth pose a challenge to traditional, in‐person active surveillance.^14^, ^15^ These factors can be expected to influence active surveillance of low‐risk skin cancers as well.

Providing effective dermatologic care through mHealth apps for older adults requires identification of cognitive, motivational and physical barriers to the use of this technology.^16^, ^17^, ^18^ While studies have identified ageing barriers and facilitators to mHealth use in older adults, it is unclear whether these factors would also influence mHealth app use for active surveillance in older dermatology patients.^17^, ^18^, ^19^, ^20^ Thus, further research is needed to better understand the intersection of barriers and facilitators regarding active surveillance and mHealth use in older adults with skin disease such as BCC.

The purpose of this study is to describe the key factors that could serve as barriers or facilitators to active surveillance of low‐risk skin cancers using mHealth technology in older adults.

In addition to exploring behaviours themselves, we intended to assess the attitudes and beliefs of participants, defined by the Theory of Planned Behavior as a person's evaluation of a behaviour of interest and appraisal of whether important peers or society would accept that behaviour, respectively.^21^ By analysing the attitudes, beliefs and behaviours of patients regarding active surveillance and mHealth, we aimed to elucidate user perspectives that could contribute to the future success of digital platforms in dermatology.

## 2 METHODS

### 2.1 Data collection

We utilized a combination of provider referrals, flyers and online community recruitment methods to identify older adults 65 years or older who have seen a dermatologist in the last 18 months. Recruitment flyers were distributed to Stanford Dermatology and Denver Tech Dermatology Clinics, with dermatologists from both locations providing referrals. Exclusion criteria included moderate‐to‐severe cognitive impairment as determined by the Mini‐Mental State Exam.^22^ In order to understand perspectives of caregivers, we also invited caregivers of patients to participate.

Two research associates with training in qualitative research methods (NS and AJ) conducted interviews in person from December 2019 to February 2020 and over video (Zoom software) from March to July 2020 due to COVID‐19 safety restrictions.

After review of the existing literature, we developed a semi‐structured interview guide that was organized by topic (general background, dermatology experience and active surveillance, and technology).^9^, ^16^, ^23^ Interviewers collected information on demographic characteristics, history of skin disease and participant opinions of hypothetical mHealth skin cancer active surveillance scenarios. While data saturation was reached before completing all interviews, we continued data collection with all potential participants because of variable geographic locations and age groups. Interviews lasted 25‐90 minutes and were audio‐recorded using encrypted voice recorders and transcribed verbatim.

### 2.2 Analysis

We conducted a thematic analysis of responses to open‐ended questions, using inductive techniques to identify key themes.^23^ Using Dedoose, we first reviewed the data to create a preliminary set of codes to capture emergent ideas within and across interviews. These codes were discussed, revised and organized into a structured codebook. Two members of the Stanford team (NS and AJ) applied the codes to a randomly assigned set of 50% of the interviews each after achieving excellent inter‐rater reliability (Cohen's kappa = 0.83). We used an iterative process to identify, refine and organize themes related to key factors influencing active surveillance and mHealth app into a mHealth for active surveillance conceptual model that groups the themes and illustrates how each could impact mHealth, active surveillance, or both domains at once.^4^ Finally, we organized all coded excerpts by their relevant themes and identified exemplary quotes illustrating how each served as a barrier or facilitator to active surveillance and/or mHealth app use. This study was approved by the Stanford Institutional Review Board.

## 3 RESULTS

Participant demographics are summarized in Table 1. A total of 33 participants were interviewed (mean age 71 years; range 56 to 92), including 4 caregivers (mean age 61; range 56 to 64). 54% of participants were female, 26 had earned at least a bachelor's degree (79%), and 97% were white. Twenty participants rated their health as very good or excellent (61%), and 21 had a previous skin cancer diagnosis (64%).

**Table 1:** Characteristics and responses of participants who completed interviews in person between December 2019 and February 2020 and through Zoom Video Software from March to July 2020 (n = 33)

Characteristic	n (%) Total n = 33
Sex	
Female	18 (54)
Race	
White	32 (97)
Asian	1 (3)
Hispanic	0 (0)
Marital status	
Now married	24 (73)
Divorced	6 (18)
Separated	1 (3)
Widowed	2 (6)
Age range	
50‐59	2 (6)
60‐69	12 (36)
70‐74	16 (48)
80+	3 (9)
Income	
N/A	10 (30)
<60 k	5 (15)
60 k‐99 k	6 (18)
>100 k	12 (36)
Education	
<Bachelor's degree	7 (21)
Bachelor's degree	13 (39)
Professional degree beyond a bachelor's/doctorate degree	13 (39)
How would you rate your health?	
Poor	1 (3)
Fair	4 (12)
Good	8 (24)
Very good	13 (39)
Excellent	7 (21)
Have you ever been told by a doctor that you had any type of cancer?	
Yes	21 (64)
No	12 (36)
How many BCCs have you had?	
N/A	9 (27)
0‐2	18 (54)
3‐7	4 (12)
8‐12	2 (6)
How much do you weigh?	
More than 130 lbs	23 (70)
130 lbs or less	10 (30)

Abbreviation: BCC, Basal cell carcinoma.

**Figure 1:**
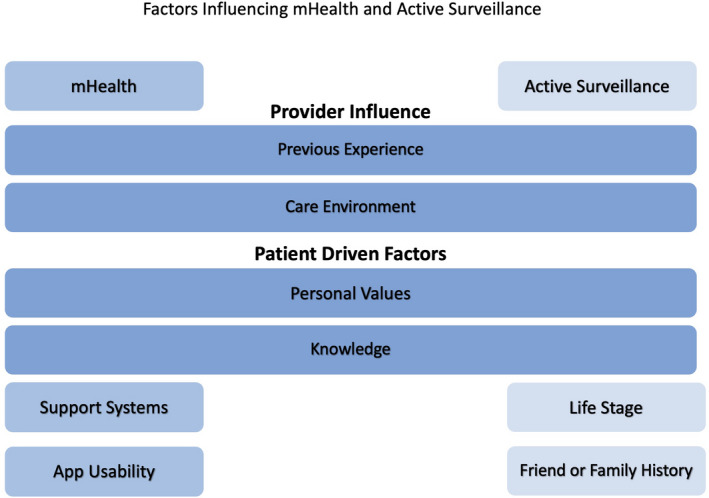
Conceptual model of themes and relationships between thematic categories derived from older adult dermatology patients regarding knowledge, attitudes and beliefs about mHealth and AS in dermatology

We identified 8 themes as facilitators, barriers or both facilitators and barriers to skin disease active surveillance or mHealth use that are summarized in the conceptual model. Our conceptual model highlights the interconnectedness of these themes and the way they influence patients' knowledge, attitudes and beliefs about active surveillance and mHealth (Figure 1). The 2 domains of active surveillance and mHealth each contain 3 thematic categories (Provider Influence, Patient Driven Factors and a thematic category unique to the domain) organized based on whether the themes were derived from provider influences, patient‐driven factors, or the domain of mHealth or active surveillance itself (Figure 1). The themes comprising the Provider Influence and Patient Driven Factors thematic categories were shared between mHealth and active surveillance. Each theme had the potential to serve as a barrier, facilitator or both to mHealth or active surveillance. For example, the *Previous Experience* theme in the Provider Influence thematic category encompasses both positive and negative participant experiences that may make patients more or less amenable to future mHealth use, respectively. Tables 2 and 3 further delineate whether each theme was a barrier, facilitator or both to mHealth or active surveillance.

**Table 2:** Barriers and facilitators to mHealth and mHealth Active surveillance use in older adult dermatology patients among 6022 qualitative excerpts, by thematic category and theme

Thematic category and Theme	Subtheme (type of factor)	Exemplary Quotes
Provider influence		
Previous experience	Facilitator	*‘I think it's pretty easy to navigate, and if the medical field can get it where people can just kind of push a button and I mean at Archuleta's (primary care) you have kind of like an iPad and it has all your information in it, you make your payment, and you go…and it's all done. So I think, you know, in that kind of application it will save them time, it will save the people at home’. Participant 32*
		*‘Bob and I are going to get hearing aids and they want to put us on the portal system that we can kind of monitor how our hearing is going to be, and I really want to do that because we're going to have rechargeable batteries so if we can learn how to do that then he can help us monitor our sound’. Participant 32*
	Barrier	*‘I have the MyHealth app, but I find that you can't get in as frequently, as close as you want to, and if I call I can usually get in, you know, a little bit sooner…than if I go on the app. But the app is great for followup… but not as good for… the scheduling is so far away that… it's not really good for me’. Participant 6*
		*‘I mean to be honest speaking, I don't use MyHealth (Stanford mHealth app) as often as I should – because I don't tend to use MyHealth to book an appointment, I still use the traditional way…I locate the doctor and I call. It seems I always get a printed copy (of the results). Because since I have a source, I have a resource…I stay with the traditional way, that's why I know it's there, just I use it (MyHealth) only when I needed to communicate with them, so if I have questions I go there and then, you know, I can write down my concerns or have the prescription refilled’. Participant 15*
Care environment	Facilitator	*‘I would be comfortable with whatever the remedy you (doctor) gave me. Yeah, that'd be great… you'll (doctor) assist me in keeping an eye on it and reporting back through the app and then I can connect with a trusted advisor just through the app – that would be great. Convenient’. Participant 13*
		*‘I think it's a great way to communicate with your physician, with your dermatologist. Without having to go in physically for a visit… It's a big deal’. Participant 24*
		*‘It would make it easy if I had to be somewhere for an extended period of time to be about to use the app to communicate with my physician, right? So that would be a bonus, I mean, you know, a plus’. Participant 25*
		*‘Saving time and money and speeding up diagnosis and communication. I mean one of the things I think is terrific that we can email our doctors and get an answer quickly. I'm all for it. It makes good sense’. Participant 11*
	Barrier	*‘The only challenge I would say is if it doesn't consume much time so it doesn't become a burden. I’m not saying it would be for me but I’m saying that generally speaking… maybe the frequency of doing it (using app)’. Participant 12*
		*‘I probably wouldn't do that… Because, first of all I live very close to Stanford. I drive here, I make an appointment, I’d have a professional look at it and do it and take care of it… Let's just get it done and move on. I don't mess around’. Participant 6*
		*‘I think it's most helpful for the physician to see it and feel it’. Participant 18*
		*‘I don't know if they're magnifying glasses that the dermatologists wear? I don't know what they can see with those that they couldn’t see from a picture. That would be my only concern. But I'm not familiar enough to know what those’. Participant 16*
Patient driven factors		
Personal values	Facilitator	*‘That would be…reaffirming, you know, that somebody cared’. Participant 10*
		*‘My only concern would be my personal incapability of using iPhones like everybody else uses them.. it's not a comfort zone for me. Not out of fear, it's just I’ve… I just have never really needed to do it. But I’m all for it… and I have no problem having a go at it, absolutely’. Participant 11*
	Barrier	*‘Probably not (open to using app). Maybe it's a generational thing, I’m not really used to using an app…I’m used to just picking up the phone. But if I had to… I would be willing to learn how to use the app… They're (apps) important, they really do help in times like this…like Zoom’. Participant 24*
		*‘I’m an old fashioned guy… It doesn't appeal to me that much… It's just another complication in an already too complicated life… You know, everybody tries to think that or believe that an electronic solution to a problem is the only way to go and is necessarily better than the old fashioned way. I can think of many instances in my life where that has not been the case’. Participant 18*
Knowledge	Facilitator	*‘Yeah, I would do that. I think it's a great idea. How long does it take to take a picture? [laughter]’ ‐ 92 yo Participant 2*
	Barrier	*‘Now I don't know why – but I have this feeling like the laptop computer is more secure than my smart phone is, but for an app what we're talking about here for the BCC…using a smart phone for that, I don't think I'd have a problem with that. I get concerned over the financial sort of stuff… So I know there's a certain amount of security and I''m just not used to it’. Participant 25*
Unique mHealth themes		
Support systems	Facilitator	*‘As long as my kids and my wife are alive, it would be okay… Because I’m not a techy guy…So I’d be okay with it if I’ve got somebody to help me’. Participant 17*
		*‘I think it would be great if you could do that (use app) and not have to get yourself to the clinic. If it seemed too complicated to do, I mean there are enough people around… There are two people in the family that are in IT and are extremely savvy and could help and can teach and can support me’. Participant 5*
		*‘If I could reach it (skin lesion). I know I can monitor my back…I do sometimes ask a massage therapist, ‘you can see better than I do, please help”’. Participant 9*
	Barrier	*‘I do feel sorry for people, older people like in their 80s and 90s that don't have a support person to help them navigate (the app). That's going to be an issue… But I think all in all, if that happens, I think it will be good for the future… It's just the simpler you can get people to navigate it, even some elderly people because a lot of elderly people are not stupid’. Participant 32*
		*It would be very difficult for me to take pictures of my back, okay, so I would have to ask my wife to do that…others may not be (able to). So I would say the location of what you're talking about, the cancer, would be dictated by who could help you or if you could do it yourself. Obviously you can't take pictures of your back, okay? Or the top of your head or whatever’. Participant 21*
App usability	Barrier	*‘Only thing is that I don't have the dexterity of…people… I don't like…texting. I don't have the feeling…like it's hard for me to turn the pages of the newspaper… These all feel like…Almost like leather. I can feel pressure…but the… the fine muscles…no, I just don't have it. That's why I will often dictate…for texts. And I get frustrated ‘cause…Siri doesn't…understand me’. Participant 9*
		*‘I don't know how to upload them onto my (cloud drive)… I mean I know how to send it to myself with an email or text, but I don't know how to directly upload it and I don't know… even really know if those are different things. [laughter]’ Participant 5*
		*‘Yeah, I can take photos and videos with my phone, but I don't use them to send to other people. I’m kind of getting a little better at it, but I’m not proficient at it – I usually have to have some help to do that… If I had to start learning how to do it I could do it, but I don't have a need for it right now. But as we talked about the waiting and watching and telemedicine, I could learn’. Participant 17*
		*‘I would worry that the picture would be of quality enough that, you know, you could actually see what it (skin lesion) is’. Participant 27*

**Table 3:** Barriers and facilitators to active surveillance in older adult dermatology patients among 6022 qualitative excerpts, by thematic category and theme

Thematic category and Theme	Subtheme (type of factor)	Exemplary Quotes
Provider influence		
Previous experience	Facilitator	*‘Well that's what I would prefer (AS), since it's not invasive it seems like there's no danger in just waiting and watching. I’ve been reluctant to have more biopsies’. Participant 28*
		*‘It sort of depends on what the cancer is and how old you are. I think right now there are great merits in watching and waiting. I’ve been in the hospital enough times, I don't need anymore!’ Participant 4*
		*‘I don't want him (dermatologist) to do anything he doesn't have to do and if it's (skin lesion) something that will resolve itself then I'm happy with that’. Participant 30*
	Barrier	*‘See I wouldn't want to do that (AS)…because I’ve had too much stuff done and, you know… I… I can't take a risk of that. I mean if I see something I have to take care of it’. Participant 6*
		*‘I'd have a poor feeling towards choosing that (AS). To me the sum of the cost that I do in monitoring versus the cost that was about the same as the cost of a hamburger, just sit there, get that (BCC) scraped, okay, it hurt a little, but I learned about how to do the procedure and I can do another one myself like we were talking about’. Participant 14*
Care environment	Facilitator	*‘No, absolutely. I’d trust their (doctors) judgment. It's their profession. I’m not gonna fight ‘em on it’. Participant 30*
		*‘I would consider it (AS). I would really want to have a good conversation with my doctor and probably do some research on it’. Participant 27*
		*‘I feel positive towards that idea of waiting and watching as long as I know somebody's (doctor) watching!’ Participant 4*
	Barrier	*‘Oh I think it's (AS) the way to go. It's acceptable under certain circumstances. Smart. Well if you don't have any…if you don't have a very aggressive (skin) situation and if you have a compliant patient! And a truthful (patient). [laughter] A lot of times people just lie about how they are really’. Participant 10*
Patient driven factors		
Personal values	Facilitator	*‘I would be comfortable with that (AS). I’d keep an eye on it myself to see if it (BCC) changed in color or dimension or it spread or something, yeah, I’d be fine with watch and wait’. Participant 13*
		*‘Well after you just described it (AS) to me, because it's like it's not like a procrastination thing, it's a helpful thing, I would probably be open to that’. Participant 19*
	Barrier	*‘I mean that (AS) to me is…The few things (skin lesions) that I’ve ever had I don't see the upside of watching and waiting…’cause I just would assume things would be worse. You know, why would you do that?’ Participant 11*
		*‘I’m pretty vigilant in terms of… What the parts of my body that I can see… Checking things and probably maybe worrying about things more than I need to!’ Participant 20*
		*‘I kind of don't have time! I just…I like to get things…I like to take care of things and be done with it’. Participant 16*
		*‘Why would I go in five times to a doctor and get an appointment and have them watch, even if it (skin lesion) isn't going anywhere? Why would I take that person's time, number one. Why would I take my time doing that, number two. What is the cost of having a scratch removal or even a slight, you know, it's not Mohs, it's just a… little incision… take that skin off, whatever… the cost and time is very low at first because… well, now that I’ve had it I’d see that it's a very low cost and so why not just…take care of it? Why? So I don't know a reason why… I don't know any negatives about taking care of it right then’. Participant 14*
Knowledge	Facilitator	*‘I think the body has like a natural recovery system within us and I would watch and wait and see, okay, is this (skin lesion) getting bigger, is it going away, so I would be fine just, if it's not bothering me and it's not spreading or swelling or inflaming, you know, no big deal to me’. Participant 13*
		*‘If you had a concern and he (doctor) said ‘I could see you in four weeks or five weeks, can you take a picture of it,’ I’d be fine with that. If I felt it (skin lesion) was explained to me that what it was was not threatening in the sense that if I don't pay attention to it, it could go south, but if it was something that was pretty minor like the pre‐cancer stuff on my hand or whatever it is and he wanted to keep an eye on it, something like that, I’d be okay with that’. Participant 17*
		*‘Probably, yeah. Probably… Well I’ve read about it (AS)… That you monitor…I mean actually my husband and I were reading something about prostate cancer and that's a new official approach to treating that – watchful waiting’. Participant 10*
		*‘I mean I’ve got dips in my nose from the surgery and stuff, so I don't know if I’m answering the question but I’m kind of…I don't know why you'd watchful wait on certain ones (skin lesions) that are bleeding maybe? Maybe the doctor should go ahead and do it (remove lesion) just to cover themselves. I’m always open to things. I’d be open to it (AS) if somebody would say, okay, if that thing's bleeding and it heals up, it's fine, you know. And maybe everybody (dermatologists) does the overboard type stuff (procedures), I don't know, I’m not educated enough to know that’. Participant 17*
	Barrier	*‘I would probably want it (BCC) removed because right now it's my belief that it would grow and it would be more disfiguring or a higher chance of it being disfiguring if… if it grew larger. If I thought that it would grow so slowly that I might die before it became a problem, then I might consider that (AS)’. Participant 9*
		*‘I think from year to year they (doctors) asked me to watch what's going on, you know…I mean and I look at that (skin lesion), you know, I mean I look when I put my makeup on, you know, and see what's…where I’m exposed and stuff, yeah’. Participant 32*
		*‘I could see where it (AS) would work on the other stuff (skin lesions) where they (doctors) can physically say, well, we're doing to freeze this or you should use cream, but the other things that are problematic when they look at it, you know, I don't know if you can watch and wait on that. I don't think’. Participant 17*
		*‘With your explanation or your definition of what that (skin cancer) is, and I know that you're talking about a slow growth or a low risk cancer is what you're talking about, I think what's problematic for me or probably for the vast majority especially at my age or younger is that…we don't recognize that as a threat or as a risk because it's slow growing and it means nothing, well that's because, you know, it's just a bump on the face or whatever it is, a rash looking thing. How would we react and seek medical attention or see a dermatologist if it's not significant to us? The likelihood that I would know that I was having a heart attack is obvious because of the signs and symptoms… I would suggest what you're describing, the signs and symptoms are so minute that we would not even recognize it until it's well advanced. I think we're missing an educational component here because you're educating me for the first time about this situation or about this technique and I could already have that form of cancer and not even know it…I guess basically you're asking me is that comforting to me if that is going to be the treatment scheme that we're going to agree with, and I’m going to say probably not because what all they're going to do is probably show me some faded diagrams or pictures, or tell me to look up a website and look at some pictures and then I’m supposed to monitor it every day when I shave or whatever. I don't take a lot of comfort in that because that's putting a bigger burden on me to determine the severity of this as opposed to the professional’*.
		*Participant 7 – ‘Well, if it's (skin lesion) a cancer. If it's a watchful waiting on a cancer I'd be…unlikely to do that. But if it's a dryness and you're, you know…(asked about what if lesion was precancer) Well that implies it's gonna turn into cancer. Why not get it off?’ Participant 21*
Unique active surveillance themes		
Life stage	Facilitator	*‘I think it (AS)would depend on my age! I mean if I’m 92 I’m not going to worry about it. In my 80s, early 80s I would probably go ahead and do it. I would probably like some information as to how the doctor felt that it (skin lesion) would progress, you know, was it dangerous to wait or…’ Participant 2*
		*‘If I’m thinking, gee, I got something else and I’m probably not gonna live more than three years, then I might say okay, let's just…watch. After age 90, I probably would be more open to that’. Participant 9*
		*‘You know what, you're getting older and it (AS) might give you four more years and that's good enough. And so that would be a time where I would choose that. This little thing on my leg, I chose right then having it. Now if it were on… somewhere where it could possibly spread, so I don't know… it's on my head, and it could spread into my brain. Well I guess I wouldn't watchful wait that’. Participant 14*
Friend or family history	Barrier	*‘I’m pretty paranoid about skin cancer because of my father, so I wouldn't be as open to that as probably if I didn't have that history… So even though his started with a mole on his back 20 years before the melanoma was full force, obviously he didn't go back and get it checked so…but hmm, I’d have to be pretty convinced… That's a tough one for me just because of the history’. Participant 16*

### 3.1 mHealth domain

#### 3.1.1 Provider influence

##### Previous experience

This theme includes participants' previous positive and negative experiences with using mHealth to access and communicate with their providers through apps and computer software. The two apps that participants referred to were Stanford and Colorado‐based primary care practice patient portals, which both offered a full range of services, including scheduling appointments, paying bills, viewing test results and communicating with a health professional. The experiences with these apps can serve as either a facilitator or barrier to mHealth use in older adult dermatology patients.

##### Care environment

This theme incorporates comments about how mHealth apps could positively impact and facilitate a participant's health care, including perceptions of quicker diagnoses and answers, easier patient‐provider communication, enhanced disease prevention surveillance, increased convenience, decreased wait time and cost and less invasiveness. Conversely, participants shared negative perceptions about mHealth and how it acted as a barrier to current or future care, including perceptions of increased time and effort, self‐preference for in‐person visits, and mistrust of quality of care when compared to in‐person visits.

#### 3.1.2 Patient driven factors

##### Personal values

This theme includes comments about participant attitudes and emotions towards mHealth use, including whether they are willing to try mHealth apps out and how mHealth does or does not align with who they are as a person. Several participants who used apps frequently were opposed to increased mHealth use, while some of those unfamiliar with technology were optimistic that they could utilize it. Thus, personal values can act as either a facilitator or barrier to mHealth use in older adult dermatology patients.

##### Knowledge

This theme captures how participants think mHealth will impact their care experience when they are unfamiliar with the technology. Some participants were hopeful that mHealth was as fast and efficient as taking a photo, while others were concerned about the security of mHealth devices.

#### 3.1.3 Unique mHealth themes

##### Support systems

This theme highlights how family or caretakers can influence participant opinions of mHealth app use. When personal support was mentioned, comments included how other family members or friends could help participants learn new technology or take photographs of hard to reach lesions. Conversely, lack of support was seen as a barrier to learning how to use mHealth apps.

##### App usability

This theme describes the individual concerns participants expressed when considering the hypothetical use of mHealth to monitor their skin condition. Participants commented on their poor physical dexterity, smartphone use, camera operation on their phone and unfamiliarity with apps that were not part of the core smartphone. Of all mHealth themes, this theme was the only factor identified to be a pure barrier to mHealth use based on participant responses.

### 3.2 Active surveillance domain

#### 3.2.1 Provider influence

##### Previous experience

This theme includes participant experiences with perceived unnecessary treatment, such as too many biopsies, surgeries and/or hospitalizations. These participants saw active surveillance as a facilitator to dermatological care that can minimize over treatment or unnecessary procedures. Conversely, significant experience with medical treatment made several participants more risk‐adverse; repeated steps to resolve a skin lesion or health condition predisposed these individuals to not want active surveillance, as they saw active surveillance as a form of risky inaction.

##### Care environment

This theme includes elements of personal patient‐provider relationships and changes to the care environment. Positive relationships and experiences with health‐care providers increased participants' trust of provider recommendations. These positive relationships seem to make participants more open to the idea of active surveillance should it be recommended to them. Conversely, weaker patient‐provider relationships, especially those in which the patient does not trust their physician or comply with her or his recommendations, are a barrier to active surveillance. In these cases, miscommunication leads participants to believe active surveillance would not work.

#### 3.2.2 Patient‐driven factors

##### Personal values

This theme captures how participants see active surveillance in the context of their personal values and attitudes, including comments on the location of the skin cancer, perceived cost of active surveillance and how active surveillance failed to align with their goals. In these cases, greater self‐confidence and awareness of how active surveillance would fit a participant's self‐identity (eg, a self‐stated procrastinator) made them more likely to consider active surveillance in a hypothetical BCC scenario. Conversely, negative attitudes or anxiety regarding a person's own health or life casts a poor light on active surveillance. Those participants who worried more about their health and future saw active surveillance as a constant contributor to their negative emotions.

##### Knowledge

This theme captures how a participant's knowledge of skin disease or active surveillance can influence how they perceive using active surveillance in their own care. Previous understanding of the low‐risk nature of BCC and how such lesions differ from other skin cancers led to more positive views towards active surveillance. In these cases, enhanced knowledge of BCC management and treatment options made participants more open to active surveillance. Conversely, incorrect beliefs about skin cancer, such as risk of BCC metastasis, or poor understanding of how active surveillance would be administered negatively impacted participants' views of active surveillance.

#### 3.2.3 Unique active surveillance themes

##### Life stage

This theme incorporated comments about a participant getting older or about their current age. Positive views towards active surveillance were expressed in actual or hypothetical cases of older age, with participants viewing active surveillance as a way to minimize overtreatment and maximize quality of life. Of all active surveillance themes, this theme was the only one to be a pure facilitator to active surveillance based on participant responses.

##### Friend or family history

This theme captures comments from participants who had a family member or friend with skin cancer. If that family member or friend either suffered or died from the disease, the participant was less inclined to consider active surveillance in a hypothetical BCC scenario. These attitudes stemmed from increased concern about a participant's own skin health, lack of knowledge of how a BCC differs from more severe skin cancers or a combination of both factors. Of all active surveillance themes, this theme was the only one identified as a pure barrier to active surveillance.

## 4 DISCUSSION

Our study reveals several key factors shaping patients' willingness to use to active surveillance using mHealth for dermatologic disease and low‐risk skin cancers. When determining whether older adults would use mHealth, it is important to consider the usability of the app, changes to the patient‐provider interaction, whether the technology aligns with a person's values and the presence or absence of support systems such as caregivers. When determining whether older adults would adopt active surveillance for low‐risk skin cancers, it is important to consider changes to the patient‐provider encounter and alignment with personal values, but a person's life stage and experience with skin cancer through close connections should also be considered. Given the overlap of these factors, it is essential to address barriers and facilitators from both domains when designing a new active surveillance approach with novel mHealth technology.

Previous studies on factors affecting active surveillance derive from the prostate cancer literature.^19^ Older age and greater medical knowledge were commonly cited facilitators to prostate cancer active surveillance, while social influences such as the wishes of family members and negative attitudes compounded by feelings of worry were found to be a barrier to active surveillance acceptance. A systematic review analysing men's choice of and adherence to active surveillance for low‐risk prostate cancer found several key factors, including patient knowledge, family influence (friend or family history) and provider characteristics (provider influence).^24^ We found similar themes to arise when older adult dermatology patients were asked whether they would consider active surveillance for low‐risk skin cancer such as BCC (Table 2). These results suggest that many of the anticipated barriers to active surveillance in dermatology can be mitigated by existing strategies used by providers in other fields when discussing active surveillance.^25^

Like in prostate cancer active surveillance, several studies have evaluated the key factors influencing mHealth adoption and use in older adults.^16^, ^17^, ^18^, ^20^, ^26^, ^27^ These studies highlight physical ability and personal values like perception of usefulness as barriers or facilitators to mHealth use in the older population.^16^, ^20^ Our findings support the influence of patient‐driven factors on mHealth app use in older adult dermatology patients (Table 1). However, we found previous mHealth experiences, support systems and perceived impacts on the patient‐provider relationship to also have the potential to act as both barriers and facilitators to mHealth use. These results suggest that applying existing older adult mHealth frameworks that omit provider and social support related factors may lead to decreased acceptance and use of mHealth in older adult patients.^16^, ^20^ Further, given the impact of COVID‐19 on the delivery of health care and projected permanence of telemedicine across specialties, it is important to consider every factor that could aid or impede mHealth use in older, higher‐risk patients.^14^, ^15^

While our framework draws on concepts found in other active surveillance and mHealth models in the literature, ^9^, ^16^, ^17^, ^20^ it is novel in its integration of the patient‐ and provider‐level factors that influence attitudes and beliefs regarding the domains of mHealth and active surveillance, which were previously analysed by separate bodies of research. Moreover, our study is the first qualitative study to analyse the intersection of mHealth and active surveillance in older adult dermatology patients. Our findings highlight similarities between the factors that influence active surveillance in other disease states such as prostate cancer and expand on previous conceptual frameworks to provide greater understanding about how barriers and facilitators to mHealth and active surveillance interact. For example, focusing on the usability of a mHealth app could result in some success, but this effort would not address issues from a patient's negative perception towards low‐risk skin cancer. Future studies should evaluate the relationships among themes identified in this conceptual model using additional methods. With further refinement, this model could provide a roadmap for anticipating potential barriers and increasing the likelihood of successfully implementing active surveillance and mHealth interventions for older adult dermatology patients.

This is the first qualitative study to examine on use of technology for active surveillance of low‐risk skin cancers. However, the findings of this analysis should be viewed as preliminary given the limitations of the methodology. We recognize this dataset comes from a highly selected voluntary sample. Those who had stronger beliefs about their dermatological experiences or were better at using technology may have been more likely to respond. We also recognize the high proportion of participants who have advanced degrees compared to the general population. However, given the increasing accessibility of mobile technology to all patients regardless of education, our findings are likely relevant to all demographics. Finally, we believe response bias is likely not a major concern because our semi‐structured interviews encompassed broad dermatology and technology‐related questions in addition to those about active surveillance and mHealth.

These preliminary findings suggest that factors influencing patient acceptance and adoption of active surveillance in dermatology would likely be similar to those in other specialties utilizing active surveillance for low‐risk cancers. Further, factors that facilitate or hinder mHealth use in older adults should be expanded to incorporate the influence of the patient‐provider relationship and presence of social support. Given the potential of active surveillance for low‐risk skin cancers to be facilitated through digital technology, it is important to understand factors that are likely to influence older patients' decisions to monitor skin conditions. As the COVID‐19 pandemic has led to a rapid adoption of telemedicine, special attention to the needs and priorities of older adults is essential to be able to deliver high quality care to all who need it.

## CONFLICT OF INTEREST

None.

## AUTHOR CONTRIBUTIONS

Austin Johnson, Neha Shukla, Meghan Halley and Eleni Linos made study concept and design. Austin Johnson, Neha Shukla, Vanessa Nava, Janya Budaraju and Lucy Zhang analysed and interpreted the data. Austin Johnson drafted the manuscript. Meghan Halley, Lucy Zhang and Eleni Linos critically revised the manuscript for important intellectual content. Austin Johnson, Vanessa Nava and Janya Budaraju contributed to statistical analysis. Eleni Linos obtained funding and study supervision.

## Data Availability

The data that support the findings of this study are available on request from the corresponding author. The data are not publicly available due to privacy or ethical restrictions.

## References

[1] Garcovich S, Colloca G, Sollena P, et al. Skin cancer epidemics in the elderly as an emerging issue in geriatric oncology. Aging Dis. 2017;8(5):643‐661. 10.14336/AD.2017.0503 28966807 PMC5614327

[2] Linos E, Chren M‐M, Stijacic Cenzer I, Covinsky KE. Skin cancer in U.S. elderly adults: does life expectancy play a role in treatment decisions? J Am Geriatr Soc. 2016;64(8):1610‐1615. 10.1111/jgs.14202 27303932 PMC5459407

[3] Rogers HW, Weinstock MA, Feldman SR, Coldiron BM. Incidence estimate of nonmelanoma skin cancer (Keratinocyte Carcinomas) in the U.S. population, 2012. JAMA Dermatol. 2015;151(10):1081‐1086. 10.1001/jamadermatol.2015.1187 25928283

[4] Linos E, Parvataneni R, Stuart SE, Boscardin WJ, Landefeld CS, Chren M‐M. Treatment of nonfatal conditions at the end of life: nonmelanoma skin cancer. JAMA Intern Med. 2013;173(11):1006‐1012. 10.1001/jamainternmed.2013.639 23699934 PMC3726204

[5] Wehner MR, Shive ML, Chren M‐M, Han J, Qureshi AA, Linos E. Indoor tanning and non‐melanoma skin cancer: systematic review and meta‐analysis. BMJ. 2012;345:e5909. 10.1136/bmj.e5909 23033409 PMC3462818

[6] Viola KV, Jhaveri MB, Soulos PR, et al. Mohs micrographic surgery and surgical excision for nonmelanoma skin cancer treatment in the Medicare population. Arch Dermatol. 2012;148(4):473‐477. 10.1001/archdermatol.2011.2456 22508870

[7] Swetter SM, Tsao H, Bichakjian CK, et al. Guidelines of care for the management of primary cutaneous melanoma. J Am Acad Dermatol. 2019;80(1):208‐250. 10.1016/j.jaad.2018.08.055 30392755

[8] Linos E, Schroeder SA, Chren M‐M. Potential overdiagnosis of basal cell carcinoma in older patients with limited life expectancy. JAMA. 2014;312(10):997‐998. 10.1001/jama.2014.9655 25203077

[9] Horrill T. Active surveillance in prostate cancer: a concept analysis. J Clin Nurs. 2016;25(7–8):1166‐1172. 10.1111/jocn.13111 26786713

[10] Taylor KL, Hoffman RM, Davis KM, et al. Treatment preferences for active surveillance versus active treatment among men with low‐risk prostate cancer. Cancer Epidemiol Prev Biomark. 2016;25(8):1240‐1250. 10.1158/1055-9965.EPI-15-1079 PMC4970911 27257092

[11] Haymart MR, Miller DC, Hawley ST. Active surveillance for low‐risk cancers — a viable solution to overtreatment? N Engl J Med. 2017;377(3):203‐206. 10.1056/NEJMp1703787 28723330 PMC5921045

[12] Parikh RR, Kim S, Stein MN, Haffty BG, Kim IY, Goyal S. Trends in active surveillance for very low‐risk prostate cancer: do guidelines influence modern practice? Cancer Med. 2017;6(10):2410‐2418. 10.1002/cam4.1132 28925011 PMC5633554

[13] Hamdy FC, Donovan JL, Lane JA, et al. 10‐year outcomes after monitoring, surgery, or radiotherapy for localized prostate cancer. N Engl J Med. 2016;375(15):1415‐1424. 10.1056/NEJMoa1606220 27626136

[14] Fisk M, Livingstone A, Pit SW. Telehealth in the context of COVID‐19: changing perspectives in Australia, the United Kingdom, and the United States. J Med Internet Res. 2020;22(6):e19264. 10.2196/19264 32463377 PMC7286230

[15] Hong Y‐R, Lawrence J, Williams D Jr, Mainous A III. Population‐level interest and telehealth capacity of US hospitals in response to COVID‐19: cross‐sectional analysis of google search and national hospital survey data. JMIR Public Health Surveill. 2020;6(2):e18961. 10.2196/18961 32250963 PMC7141249

[16] Wildenbos GA, Peute L, Jaspers M. A framework for evaluating mHealth tools for older patients on usability. Stud Health Technol Inform. 2015;210:783‐787. 10.3233/978-1-61499-512-8-783 25991261

[17] Wildenbos GA, Peute L, Jaspers M. Aging barriers influencing mobile health usability for older adults: a literature based framework (MOLD‐US). Int J Med Inf. 2018;114:66‐75. 10.1016/j.ijmedinf.2018.03.012 29673606

[18] Wildenbos GA, Jaspers MWM, Schijven MP, Dusseljee‐Peute LW. Mobile health for older adult patients: using an aging barriers framework to classify usability problems. Int J Med Inf. 2019;124:68‐77. 10.1016/j.ijmedinf.2019.01.006 30784429

[19] Pereira‐Azevedo NM, Venderbos LDF. eHealth and mHealth in prostate cancer detection and active surveillance. Transl Androl Urol. 2018;7(1):170‐181. 10.21037/tau.2017.12.22 29594031 PMC5861289

[20] Spann A, Stewart E. Barriers and facilitators of older people's mHealth usage: a qualitative review of older people's views. Hum Technol. 2018;14(3):264‐296. 10.17011/ht/urn.201811224834

[21] LaMorte WW. Behavioral Change Models. https://sphweb.bumc.bu.edu/otlt/MPH‐Modules/SB/BehavioralChangeTheories/BehavioralChangeTheories_print.html. Accessed January 11, 2021.

[22] McKhann GM, Knopman DS, Chertkow H, et al. The diagnosis of dementia due to Alzheimer's disease: recommendations from the National Institute on Aging‐Alzheimer's Association workgroups on diagnostic guidelines for Alzheimer's disease. Alzheimers Dement J Alzheimers Assoc. 2011;7(3):263‐269. 10.1016/j.jalz.2011.03.005 PMC3312024 21514250

[23] Miles M, Huberman AM, Saldana J. Qualitative data analysis: A methods sourcebook, 4th edn: SAGE Publications; 2020. https://us.sagepub.com/en‐us/nam/qualitative‐data‐analysis/book246128

[24] Kinsella N, Stattin P, Cahill D, et al. Factors influencing men's choice of and adherence to active surveillance for low‐risk prostate cancer: a mixed‐method systematic review. Eur Urol. 2018;74(3):261‐280. 10.1016/j.eururo.2018.02.026 29598981 PMC6198662

[25] Xu J, Dailey RK, Eggly S, Neale AV, Schwartz KL. Men's perspectives on selecting their prostate cancer treatment. J Natl Med Assoc. 2011;103(6):468‐478. 10.1016/s0027-9684(15)30359-x 21830629 PMC4283563

[26] Rittenmeyer L, Huffman D, Alagna M, Moore E. The experience of adults who choose watchful waiting or active surveillance as an approach to medical treatment: a qualitative systematic review. JBI Database Syst Rev Implement Rep. 2016;14(2):174‐255. 10.11124/jbisrir-2016-2270 27536798

[27] Lefler LL, Rhoads SJ, Harris M, et al. Evaluating the use of mobile health technology in older adults with heart failure: mixed‐methods study. JMIR Aging. 2018;1(2):e12178. 10.2196/12178 31518257 PMC6715011

